# In vivo positron emission tomographic blood pool imaging in an immunodeficient mouse model using 18F-fluorodeoxyglucose labeled human erythrocytes

**DOI:** 10.1371/journal.pone.0211012

**Published:** 2019-01-25

**Authors:** Jung W. Choi, Mikalai Budzevich, Shaowei Wang, Kenneth Gage, Veronica Estrella, Robert J. Gillies

**Affiliations:** 1 H. Lee Moffitt Cancer Center and Research Institute, Diagnostic Imaging, Tampa, Florida, United States of America; 2 H. Lee Moffitt Cancer Center and Research Institute, Department of Cancer Physiology, Tampa, Florida, United States of America; 3 University of South Florida, Department of Medical Engineering, Tampa, Florida, United States of America; 4 H. Lee Moffitt Cancer Center and Research Institute, Program in Cancer Biology and Evolution, Tampa, Florida, United States of America; Wayne State University, UNITED STATES

## Abstract

99m-Technetium-labeled (^99m^Tc) erythrocyte imaging with planar scintigraphy is widely used for evaluating both patients with occult gastrointestinal bleeding and patients at risk for chemotherapy-induced cardiotoxicity. While a number of alternative radionuclide-based blood pool imaging agents have been proposed, none have yet to achieve widespread clinical use. Here, we present both in vitro and small animal in vivo imaging evidence that the high physiological expression of the glucose transporter GLUT1 on human erythrocytes allows uptake of the widely available radiotracer 2-deoxy-2-(^18^F)fluoro-D-glucose (FDG), at a rate and magnitude sufficient for clinical blood pool positron emission tomographic (PET) imaging. This imaging technique is likely to be amenable to rapid clinical translation, as it can be achieved using a simple FDG labeling protocol, requires a relatively small volume of phlebotomized blood, and can be completed within a relatively short time period. As modern PET scanners typically have much greater count detection sensitivities than that of commonly used clinical gamma scintigraphic cameras, FDG-labeled human erythrocyte PET imaging may not only have significant advantages over ^99m^Tc-labeled erythrocyte imaging, but may have other novel blood pool imaging applications.

## Introduction

Clinical blood pool imaging is commonly performed in nuclear medicine departments using autologous human erythrocytes labeled with the radiotracer 99m-Technetium (^99m^Tc) pertechnetate and gamma scintigraphic imaging. The current prevalent clinical indications for ^99m^Tc-labeled erythrocyte imaging are the detection of drug-induced cardiomyopathy in cancer patients undergoing potentially cardiotoxic chemotherapy, and anatomic localization of sites of occult lower intestinal bleeding in patients [1–3]. While planar imaging of ^99m^Tc-labeled erythrocytes is the most commonly utilized nuclear medicine blood pool imaging test, ECG-gated single-photon emission computed tomographic (SPECT) imaging of ^99m^Tc-labeled erythrocytes has also been used for radionuclide ventriculography, demonstrating comparable results to planar blood pool imaging [4–6]. In addition, blood pool imaging with magnetic resonance imaging can be performed using long-circulating gadolinium-based magnetic resonance contrast agents such as Gadofosveset and Ferumoxytol, and have been described in the evaluation of peripheral vascular disease and other specific vascular pathologies, including deep venous thrombosis, thoracic venous outlet syndrome and pelvic congestion syndrome [7].

As modern clinical PET scanners have a ≥100-fold higher count detection sensitivity over typical clinical planar scintigraphic cameras, as well as higher image quality and temporal resolution, the development of PET-based blood pool imaging agents remains of great interest [8]. A number of PET specific radiotracers have been investigated as blood pool imaging agents, but are not yet available for clinical use [9–11]. There are a few PET-specific blood perfusion agents based on 15-Oxygen-labeled (^15^O) compounds such as H_2_^15^O that are clinically available, but are unlikely to achieve wide-spread clinical adoption, as the short radioactive half-life of ^15^O (≈ 2 minutes) requires that the PET scanner be in close proximity to a cyclotron [12–15]. Other PET or SPECT blood perfusion agents have either a narrowed or incompletely characterized range of application, such as 62-Copper-pyruvadehyde-bis(N^4^-methylthiosemicarbazone) (^62^Cu-PTSM), 68-Gallium-labeled microspheres, or the ^99m^Tc-labeled cerebrovascular perfusion agents hexamethylpropyleneamine oxime (HMPAO) and ethylcysteinate dimer (ECD) [12–28].

Existing literature supports the idea that human erythrocytes can physiologically internalize a relatively large amount of the PET tracer 2-deoxy-2-(^18^F)fluoro-D-glucose (FDG) that would be sufficient for PET-based in vivo erythrocyte imaging, for a few reasons: 1) Of all cells in the human body, human erythrocytes have the highest level of expression of the glucose transporter GLUT1, with each erythrocyte expressing roughly 2–3 x 10^5^ copies of GLUT1 on the plasma membrane, 2) Human erythrocytes also express other glucose transporters, albeit at lower levels, including glucose transporter 4 (GLUT4) and Sodium-glucose co-transporters, and 3) Intracellular glucose concentrations in human erythrocytes can be high, as it typically mirrors that of human serum (4–6 mM) [29,30].

Here, we present evidence that human erythrocytes can be sufficiently labeled with FDG to acquire whole body images of the vasculature of splenectomized immunodeficient mice using a small animal positron emission tomography/computed tomography (PET/CT) scanner. In addition, the cell labeling technique is straightforward to perform and can be completed in a relatively short time period.

## Materials and methods

Unless otherwise specified, all chemicals were cell culture grade and obtained from Sigma Aldrich, St. Louis MO. Packed human erythrocytes collected in standard anticoagulant citrate dextrose (ACD) solution (either ≤ 24 hours post phlebotomy or ~ 5 day post phlebotomy) were obtained from Zen-Bio, Inc. 370–740 Megabecquerel (MBq) (1 ml) United States Pharmacopeia (USP) grade 2-deoxy-2-(^18^F)fluoro-D-glucose (FDG) were obtained from Cardinal Health. Vendor supplied human erythrocytes were centrifuged 1000g 10 minutes, and the remaining anticoagulant and buffy coat residual were gently aspirated. Erythrocytes were then gently washed in a 4X volume of filter sterilized “1X EDTA” solution (140 mM NaCl, 4 mM KCl, 2.5 mM Ethylenediaminetetraacetic acid dipotassium salt dihydrate (K_2_EDTA dihydrate)), centrifuged at 1000 x g for 10 minutes, and the wash was manually aspirated. 150 μl 1X EDTA solution was then added to the 250 μl washed erythrocytes and 100 μl (37–74 MBq) FDG to a final volume of 500 μl. For experiments using 500 μl packed erythrocytes, 400 μl 1X ETDA solution and 100 μl (37–74 MBq) FDG were added to a final volume of 1000 μl. Samples were then gently agitated on a platform rotator at either room temperature (~25° C) for 2 hours or at 37° C for 30 minutes to 2hours. Samples were then centrifuged at 1000 x g for 10 minutes and the supernatant was carefully aspirated. Erythrocytes were then washed and centrifuged twice with 4X volumes of 1X EDTA solution. For experiments characterizing residual unincorporated FDG, a 3^rd^ wash/centrifugation step was performed. Final washed FDG-labeled erythrocytes were resuspended in 1X volume of 1X EDTA solution. Aliquots from all samples and washes were counted with the Atomlab 500 dose calibrator [Biodex Medical Systems, Inc].

To measure FDG leakage from labeled erythrocytes, freshly phlebotomized venous blood from 4–6 month old male F344 (CDF) rats [Charles River] were collected and labeled with FDG using the previously described FDG labeling protocol. 250 μl of washed FDG-labeled rat erythrocytes were then incubated with 375 μl of rat plasma at 37° C for 30, 60, and 90 minutes. The samples were then centrifuged 1000g for 10 minutes, and counted with the Atomlab 500 dose calibrator. When accounting for additional time passage during the centrifugation step, sample activity measurements were made approximately 46 minutes, 76 minutes and 106 minutes after start of plasma incubation.

To evaluate the degree of mechanical erythrocyte membrane damage induced by the centrifugation and washing steps in the cell labeling process, fresh rat venous blood samples were subjected to the previously described FDG erythrocyte cell labeling protocol, but with substitution of 1X EDTA buffer solution for FDG. The erythrocytes were then incubated with acetoxymethyl ester of calcein (calcein AM), a fluorescent marker of cell membrane integrity and viability [31]. Erythrocytes were also incubated with Annexin-V-phycoerythrin (Annexin V-PE), a fluorescent marker of early eryptosis [32]. In particular, 0.5–1.0 ml of venous blood was collected from 4–6 month old male F344 (CDF) rats [Charles River] into heparinized phlebotomy tubes. After mock FDG labeling of 250 μl of packed rat erythrocytes, approximately 4 x 10^5^ rat erythrocytes in 40 μl 1X distilled phosphate buffered saline pH 7.4 were incubated in 10 μl Annexin V-PE [BD Biosciences], 80 μM calcein AM dye, and 40 μl 1X Annexin V Binding Buffer [BD Biosciences] to a final volume of 100 μl at 37° C for 30 minutes in the dark. As control groups, unperturbed rat erythrocytes or unperturbed rat erythrocytes then treated with 20 mM CaCl_2_ were stained with both calcein AM and Annexin V-PE. Rat erythrocytes were then evaluated with flow cytometric analysis at the Moffitt Cancer Center (MCC) flow cytometry core facility.

4–6 week old male splenectomized NOD *scid* gamma (NSG) immunodeficient mice were obtained from The Jackson Laboratory. Mice were placed in specialized housing for immunodeficient mice in the MCC vivarium. Approval for all animal experimentation was first obtained from the University of South Florida (USF) Institutional Animal Care and Use Committee (IACUC). All animal experimentation was performed in accordance to federal regulations and USF IACUC principles and procedures. Animal imaging was performed in the MCC Small Animal Imaging Laboratory with an Inveon PET/CT [Siemens Medical Inc., Knoxville, Tennessee] preclinical scanner. Mice were either injected with FDG-labeled human erythrocytes (4 mice) or were injected with intravenous FDG (4 mice) prior to PET imaging. Mice were first fasted the night before microPET imaging and underwent phlebotomy of ≈ 200–250 μl whole blood via retro-orbital venous plexus puncture immediately prior to microPET imaging. Blood glucose from the phlebotomized blood was measured. A tail vein microcatheter was placed in each mouse under inhalational isoflurane anesthesia. Mice were maintained under inhalational anesthesia and immobilized on the microPET platform before injection of 500 μl of FDG-labeled erythrocyte suspension through the catheter. The PET portion of the system has 64 detector blocks with a total ield of view (FOV) of 12.7 cm and a spatial resolution of 1.4 mm. Raw PET data were acquired for 10 minutes in list-mode data format, followed by the CT attenuation correction scan. Imaging was started immediately after tail-vein injection of -FDG infused erythrocytes. The ECG signal in each mouse was detected with three flat electrodes placed on two front limbs and one hind limb of each animal (ground lead on a rear leg). The signals detected by these electrodes were recorded during the 10 minute time period by BioVet [m2m Imaging] physiological monitoring and heating system. The threshold for TTL cardiac gating signals was set in a rising mode of R-wave peak. The PET list-mode data was reconstructed using 3D-OSEM iterative algorithm with four iterations and eight subsets, with a final image volume of 256x256x256 voxels. Voxel effective dimensions were 1.4x1.4x1.4 mm. For each animal there were three data sets: standard 3-dimensional (3D) PET reconstruction, resulting in a motion—time average 3D PET image; dynamic 3D PET reconstruction with 30 frames; and the phase-based 4-dimensional (regular 3-dimensional plus time, 4D) PET cardiac reconstruction, with four cardiac gate binning. In all cases, CT attenuation correction was applied to the PET images.

PET images of the mice were analyzed using Inveon Workstation Software [Siemens Medical Inc., Knoxville, Tennessee]. Vendor software-supplied Patlak compartment plot option was selected [33]. For 3D PET and 4D PET data sets, multiple volumes of interest (VOI) were selected manually, based on corresponding CT images including: heart, leg muscle, liver, kidney and brain. Voxel activities were represented in standardized uptake values (SUV). Dynamic activity curves were plotted for VOIs using dynamic 3D PET data set for each animal. The 4D PET data were used for defining cardiac function. First, the heart was segmented on CT images based on anatomical features; afterwards, the segmented volume (cardiac PET VOI) was transferred into co-registered PET images. All images in the manuscript represent maximum intensity projection (MIP) reconstructions of the source data. With regards to images of the mouse head and neck, the whole body blood-pool activities were first segmented using SUVs in a range of 0.9–7.5. The resulting contoured data was then combined with MIP data render to improve small vessel conspicuity.

All mice in the protocol are evaluated daily in the vivarium to assure well being where their behavior was observed for any signs of distress or illness. A few mice experienced transient distress during microPET image acquisition as perceived by intra-procedural tachypnea and tachycardia, during which inhalational anesthesia was carefully monitored and continuously titrated to reduce any perceived discomfort. These mice were also carefully monitored immediately after PET imaging, and noted to have quickly recovered from previous tachypnea and did not display signs of obvious discomfort, such as labored breathing or impaired movements. Immediately after imaging, mice were placed under a heating lamp and monitored for signs of distress for 1 hour. Breathing was monitored visually and recovery from inhalational anesthesia was assessed by response to sternal rubbing. After completion of the study, mice were euthanatized with an overdose of inhalational anesthesia, and death was verified by cessation of cardiovascular and respiratory movements during prolonged observation of at least 10 minutes, in accordance with USF IACUC protocol.

The FDG radiolabeled erythrocytes were assumed to distribute uniformly throughout the blood pool within a few minutes of injection (“well-mixed” assumption). Dosimetry calculations were made under two scenarios. In the first scenario, it is assumed that there is no appreciable efflux of erythrocyte internalized FDG into the plasma during imaging and beyond. In essence, there is no assumed excretion and the entire injected dose is therefore considered to undergo physical decay within the model. It was also assumed there is no significant variation in blood pool specific activity related to decay while in transit to various organ sites. These assumptions result in a static biodistribution of radiotracer throughout the body, with the organ fraction of the injected dose being equal to the organ blood volume. In the second scenario, dosimetry values were calculated under conditions of poor FDG labeling of human erythrocytes, leading to a 25% urinary excretion of released/free FDG in vivo. The values for the organ blood volumes were obtained from ICRP Publication 89 [34].

Human organ absorbed doses were estimated using the FDA approved OLINDA/EXM 1.1 [Vanderbilt University, TN] dosimetry software which contains several idealized human and tumor phantoms spanning both genders and various age groups [35,36]. Dose estimates were generated using the included reference adult male, based on the idealized Christy-Eckerman phantom [37].

The Christy-Eckerman phantom (and therefore OLINDA/EXM 1.1) assumes the self-dose to the hollow organs (e.g. the gastrointestinal tract) arises from radioactive material within the lumen (i.e., ingested dose) rather than the viscus wall itself as would be expected with a blood pool agent. The assumption results in an erroneous calculation of the electron self-dose that can be corrected [38].

## Results

**Fig 1:**
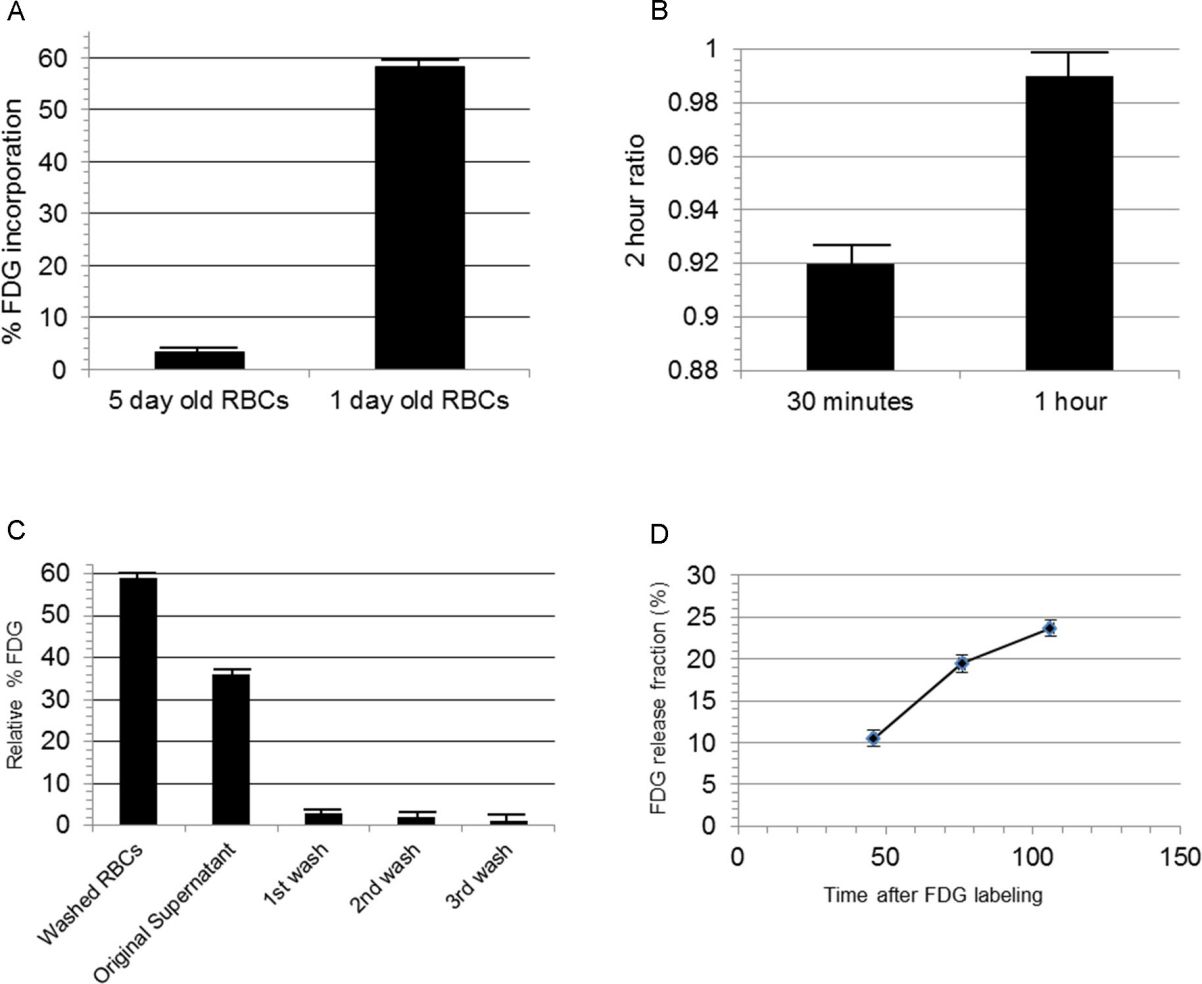
Characterization of FDG labeling of human erythrocytes. (A) FDG uptake by 5 day old and 1 day old human erythrocytes.The relative percent FDG uptake by 5 days old human erythrocytes (post-phlebotomy) vs.1 day old human erythrocytes. Either 250 μl of 1 day old human erythrocytes or 5 day old erythrocytes were incubated with 100 μl (≈ 37MBq) 18F-FDG for 2 hours at 37° C. (B) Human erythrocyte uptake of FDG after 30 minute incubation approaches that of 2 hour incubation. 250 μl of 1 day old human erythrocytes were incubated with 100 μl (≈ 37MBq) FDG at 37° C for 30 minutes, 1 hour, and 2 hours. Sample number = 3/timepoint. **(**C) Relative % free FDG remaining in the FDG-labeled erythrocyte fraction (250 μl), original incubation supernatant, and wash supernatants. (D) Slow gradual release of intracellular FDG over time (R^2^ = 0.9586; sample number = 3/timepoint) (S1 File).

We examined FDG uptake efficiency of human erythrocytes either 1 day or 5 days after phlebotomy. The percent of FDG uptake by human erythrocytes collected ≤ 24 hours prior to FDG labeling was significantly higher than that of erythrocytes collected ≈ 5 days prior to FDG labeling (Fig 1A). The mean % total FDG incorporation of 250 μl of 1 day old erythrocytes (120 minute incubation at 37° C with 37–74 MBq FDG) was 58.2% ± 0.3% (N = 5), compared to 3.4% ± 0.2% FDG incorporation of 5-day- old erythrocytes (mean ± standard error; Sample number = 6). Unpaired t test P value < 0.0001; R^2^ = 0.997.

FDG uptake by human erythrocytes was then measured over a time period of up to 2 hours. 250 μl of 1 day old human erythrocytes were incubated with 37–74 MBq FDG at 37° C at 30 minutes, 1 hour, and 2 hours. The 30 minute:2 hour FDG incorporation ratio = 0.92 ± 0.02. The 1 hr:2 hr FDG incorporation ratio = 0.99 ± 0.02 (Fig 1B). (mean ± standard error; sample number = 3)

Unincorporated FDG was then measured during each step of the cell labeling procedure. The FDG activity in the washed cell samples (500 μl), original incubation solution, and subsequent 3 washes was measured and the relative % of total FDG were as follows: Washed erythrocyte fraction (500 μl) = 59% ± 1%; Original supernatant (unincorporated FDG after initial incubation) = 36% ± 2%; 1^st^ wash supernatant = 3% ± 1%; 2^nd^ wash supernatant = 2% ± 1%; 3^rd^ wash supernatant = 1% ± 1% (Fig 1C). (mean ± standard error; sample number = 4)

To evaluate the amount of intracellular FDG released from erythrocytes over time, fresh rat erythrocytes were labeled with FDG as before (Fig 1D). Labeled erythrocytes were then incubated in rat plasma at 37° C over time. Erythrocytes were centrifuged again and a fraction of the supernatant and cell pellet were counted to calculate the relative amount of intracellular FDG leakage. Relative FDG leakage at the following time points were as follows: 46 minutes = 10.5% ± 0.5%; 76 minutes = 19.4% ± 0.9%; 106 minutes = 23.7% ± 1.1% (Average of 3 samples per time points ± SEM).

**Fig 2:**
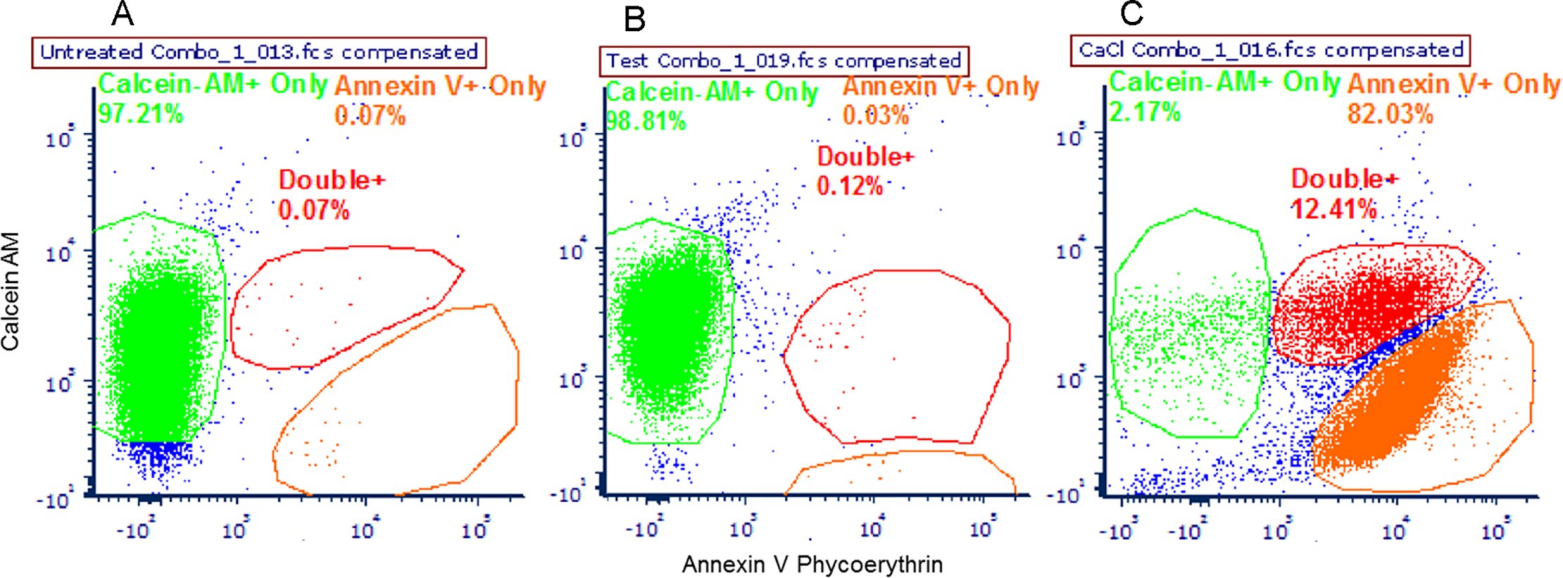
Minimal erythrocyte membrane damage is seen after repeated centrifugation and wash steps of cell labeling technique. (A) Unperturbed (negative) control group. (B) Mock FDG labeled cell group. (C) CaCl_2_-treated group. Each figure is representative of 3 samples per group.

To assess mechanical erythrocyte membrane damage induced by the repeated centrifugation and washing steps required for FDG labeling, freshly phlebotomized rat blood was subjected to the same cell labeling protocol, except for replacement of FDG with an equal volume of 1X EDTA buffer. Rat erythrocytes were used instead of human erythrocytes, due to the ready availability of fresh phlebotomy samples of sufficient volumes. After mock FDG labeling, centrifugation and washing, the rat erythrocytes were then incubated with both the cell membrane integrity/cell viability dye calcein-AM and with the early eryptosis marker Annexin V-PE. Samples were then analyzed by flow cytometry to characterize any potential damage induced by the cell labeling protocol. In comparison to the unperturbed (negative control) group (Fig 2A), the samples subjected to the cell labeling protocol experienced a tiny relative increase (< 1%) in the cell fraction expressing high levels of Annexin V-PE or low levels of intracellular calcein AM (Fig 2B). Approximately 98% of erythrocytes subjected to the cell labeling protocol demonstrate no evidence of significant membrane damage, as deemed by high calcein AM/low Annexin V-PE fluorescence. By contrast, rat erythrocytes exposed to CaCl_2_, an known apoptosis inducer, demonstrated significantly decreased cell fractions retaining high intracellular levels of calcein AM (≤2%), and had a much larger cellular fraction expressing high levels of Annexin V-PE binding (≈ 94–98%) (Fig 2C) (S1–S9 Figs).

**Fig 3:**
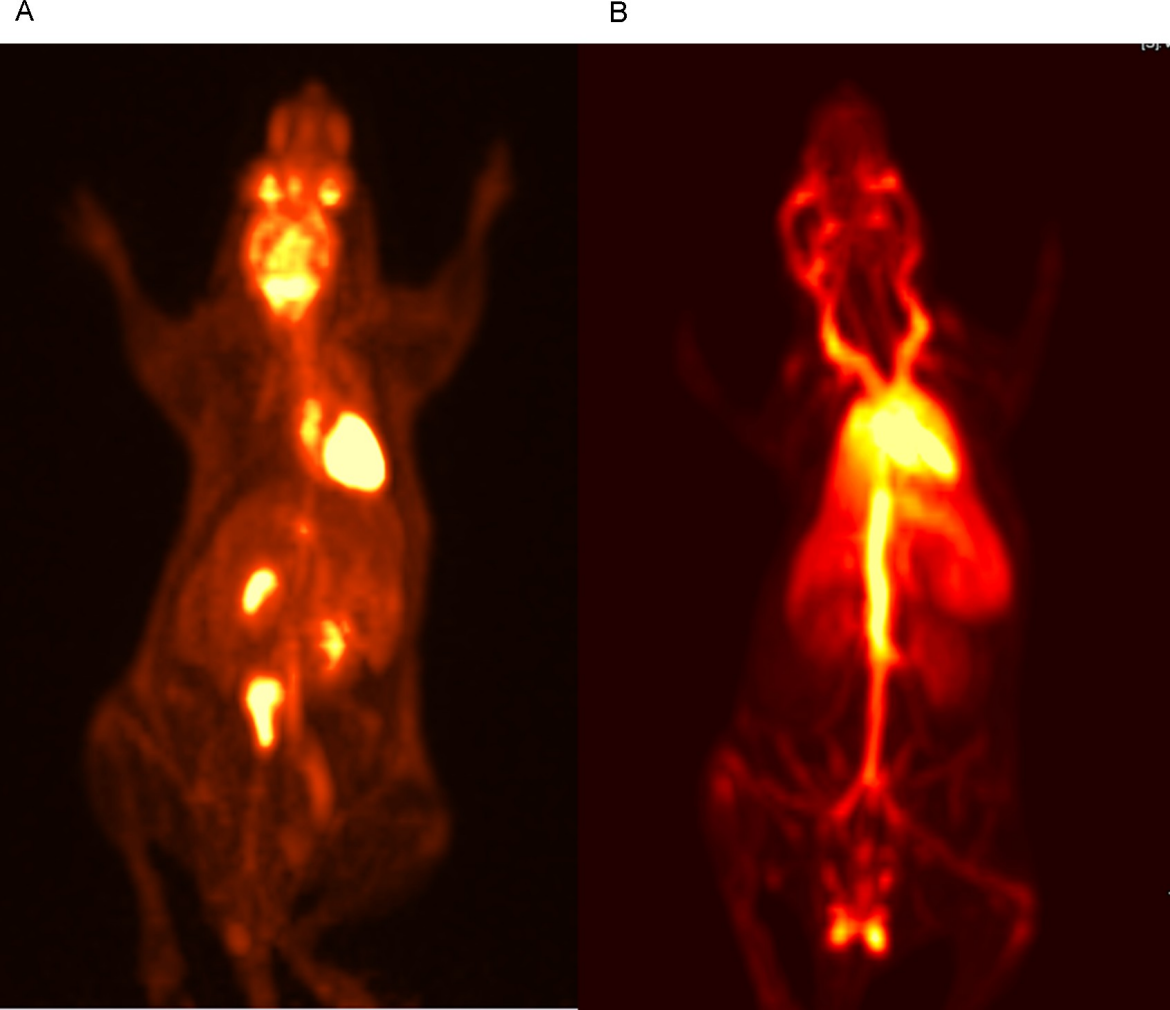
Whole body microPET images of control FDG injected mouse and mouse injected with FDG-labeled human erythrocytes. (A) Whole body PET image of a control splenectomized NSG mouse injected with 1.7 MBq of free FDG. 3(B) Whole body ECG-gated PET image of a splenectomized NSG mouse injected with 10.4 MBq of 18F-FDG-labeled human erythrocytes. Number of mice in FDG-erythrocyte injected mouse group = 4.

Splenectomized NSG immunodeficient mice were then injected with either 1.7–10.4 MBq FDG-labeled erythrocytes or 1.4–18 MBq of free FDG, and underwent ECG-gated microPET/CT imaging. The biodistribution of free FDG in control mice on whole body microPET images demonstrated expected intense FDG accumulation in the myocardium and brain, as well as marked urinary excretion in the renal pelvises and bladder (Fig 3A). The microPET images of mice injected with FDG-labeled erythrocytes show that the FDG activity was largely confined to the large vessels of the mouse body. Activity over the heart was also observed, as well as perfusion of the lungs, liver, spleen, kidneys, and the testes in male mice (Fig 3B). FDG activity within the kidneys of these mice demonstrates a predominantly vascular distribution pattern, unlike the accumulation of excreted FDG in the renal pelvises of control mice injected with free FDG. In addition, a small amount (≈5%) of total body FDG activity was seen overlying the bladder in mice injected with FDG-labeled erythrocytes, consistent with small urinary excretion of residual free/released FDG in these mice over the observed time period. There was also a relative paucity of FDG activity in the brain of these mice when compared to the control mice.

**Fig 4:**
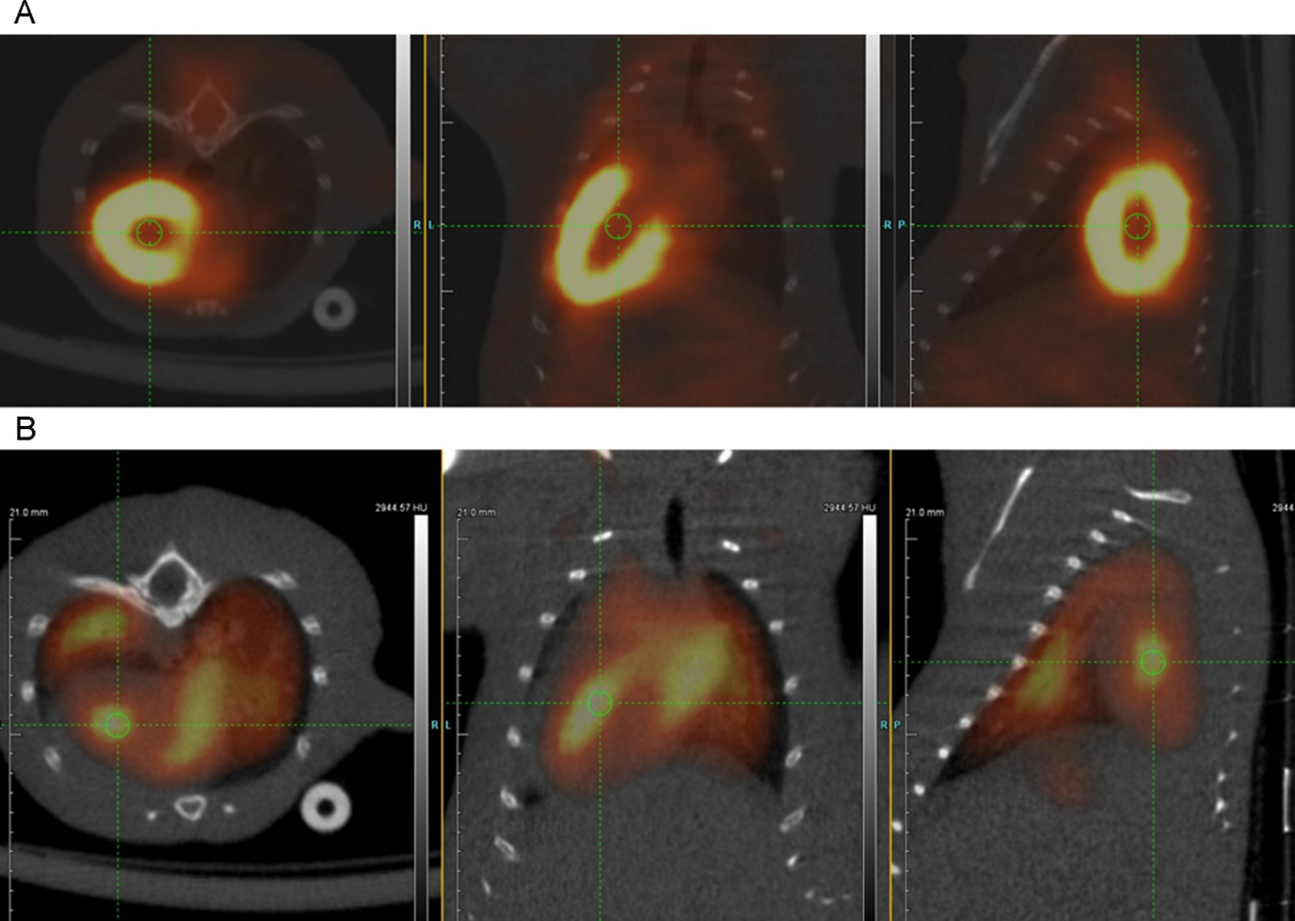
Fused PET/CT images of thorax of control FDG injected mouse and mouse injected with FDG-labeled human erythrocytes. (A) Axial (left), coronal (middle), and sagittal (right) fused microPET/CT images of the mouse thorax show intense physiologic FDG uptake by the myocardium of the left ventricle in a splenectomized NSG mouse injected with 2.2 MBq of free FDG. Green circle indicates lumen of the left ventricle. (B) Axial (left), coronal (middle), and sagittal (right) fused microPET/CT images of the mouse thorax show FDG activity within the lumen of the cardiac chambers (green circles) in a splenectomized NSG mouse injected with 1.7 MBq of FDG-labeled human erythrocytes. Pulmonary perfusion about the heart is also visualized.

Fused microPET/CT images of the heart from these mice showed clear differences in anatomic distribution of FDG in the heart (Fig 4). While marked myocardial uptake of FDG in the left ventricle of control mice was clearly visualized (Fig 4A), FDG distribution in the FDG-labeled erythrocyte injected mice showed that the activity was essentially confined to the intraluminal chambers of the heart (Fig 4B). Pulmonary perfusion about the heart was also seen in these mice, unlike control mice.

**Fig 5:**
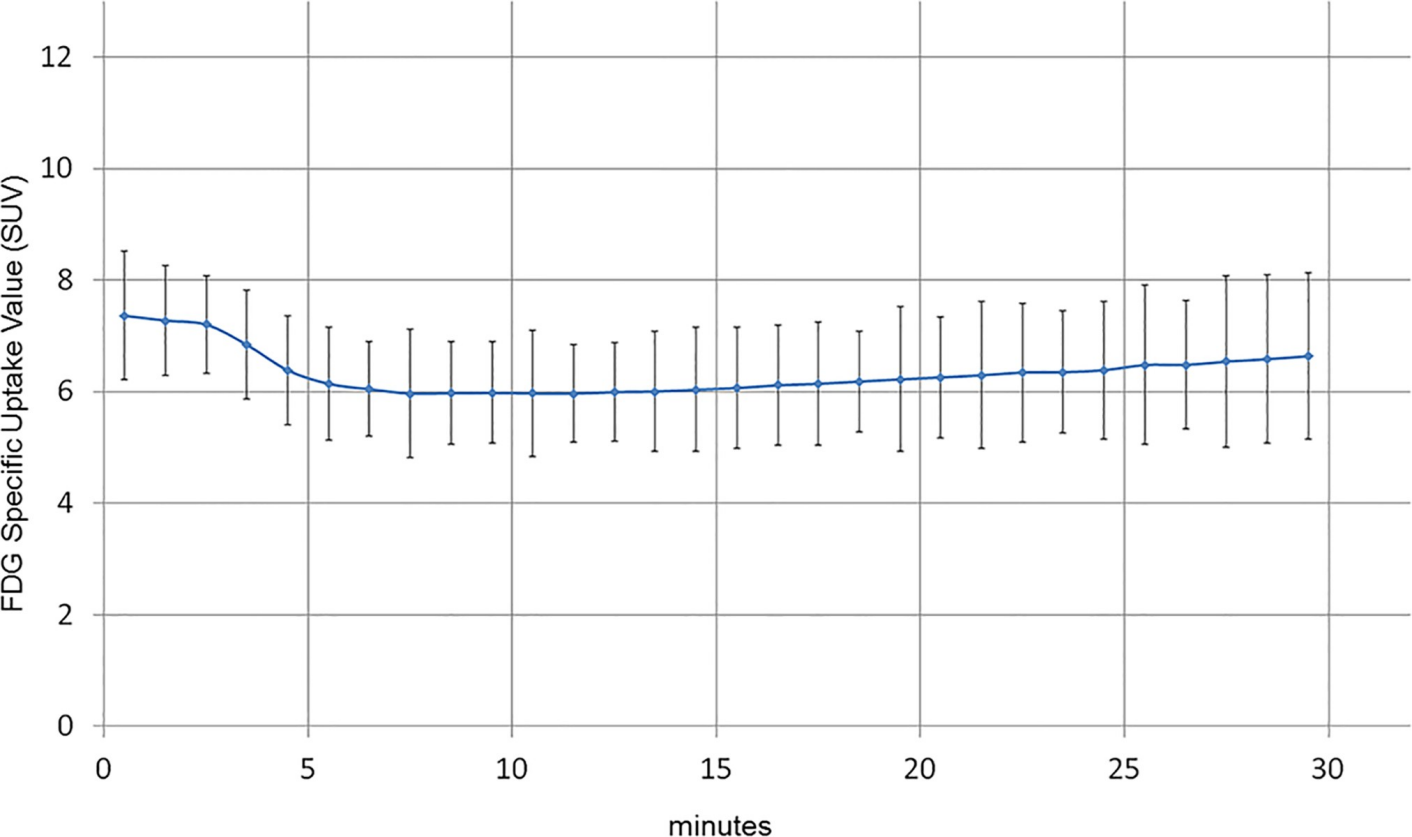
Time activity curve of mouse injected with FDG-labeled human erythrocytes. TAC of the heart of a mouse injected with FDG-labeled erythrocytes shows expected blood pool activity of FDG-labeled human erythrocytes with immediately high FDG activity (≤1 minute) followed by a small initial decline (4–14 minutes) and then activity plateau (14–30 minutes).

In the time-activity-curves of the heart from FDG-labeled erythrocyte injected mice, there was immediate high activity over the heart within 1 minute, followed by a small initial decline, then a plateau over most of the measured time period (Fig 5). In contrast, time-activity curves of the heart from mice injected with free FDG showed a steady increase of activity over the heart over the same time period, consistent with gradual myocardial uptake/accumulation of FDG (not shown). FDG activity over the heart of erythrocyte injected mice did not decline progressively over the observed time period to suggest significant in vivo erythrocyte hemolysis and urinary excretion of released FDG in these mice. In addition, ECG-gated images from erythrocyte injected mice could be rebinned according to the cardiac cycle to create cine images of cardiac motion in these mice.

**Fig 6:**
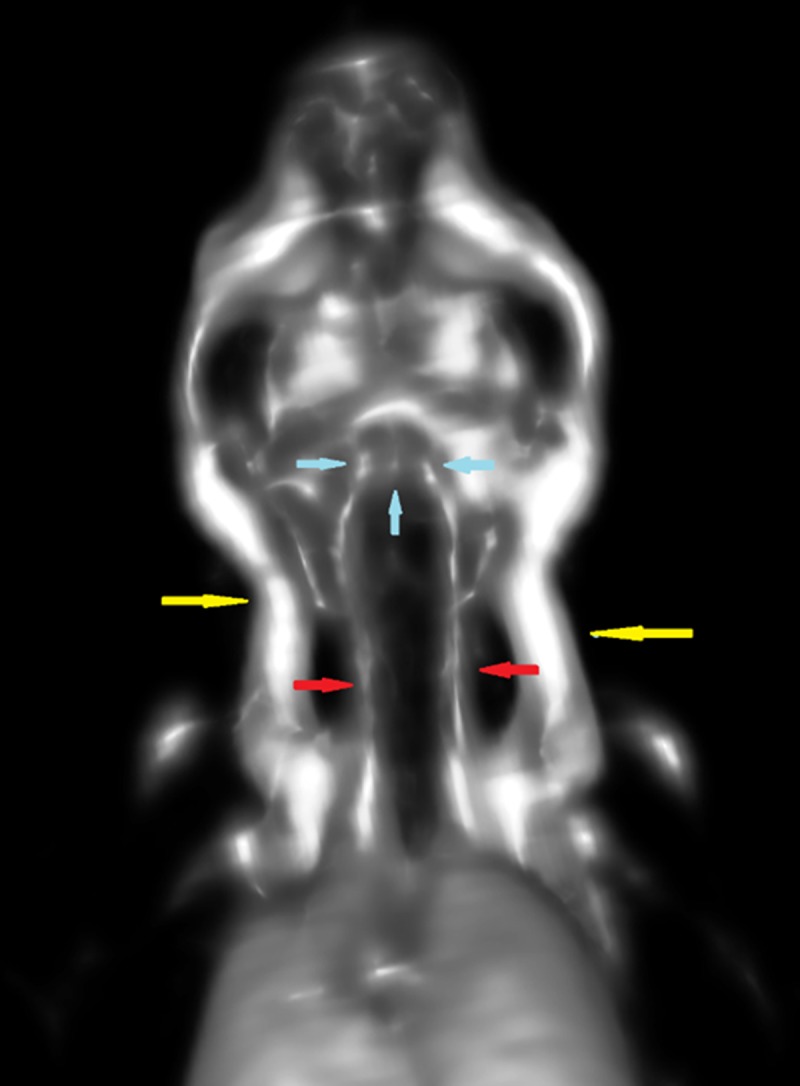
MicroPET images of the mouse head and neck allow visualization of major head and neck vessels. Major vessels in the neck of the mice injected with FDG-labeled erythrocytes could be visualized, such as the external jugular veins in the lateral neck (yellow arrows), the smaller common carotid arteries in the central neck (red arrows), and the circle of Willis (blue arrows).

Magnified views of the head and neck of the FDG-labeled erythrocyte injected mice demonstrate that FDG activity could be localized to the major head and neck vessels. These include the external jugular veins, the common carotid arteries, and the circle of Willis (Fig 6). In addition, other venous structures in the head could be visualized, such as the retro-orbital venous plexi and the transverse venous sinuses.

**Fig 7:**
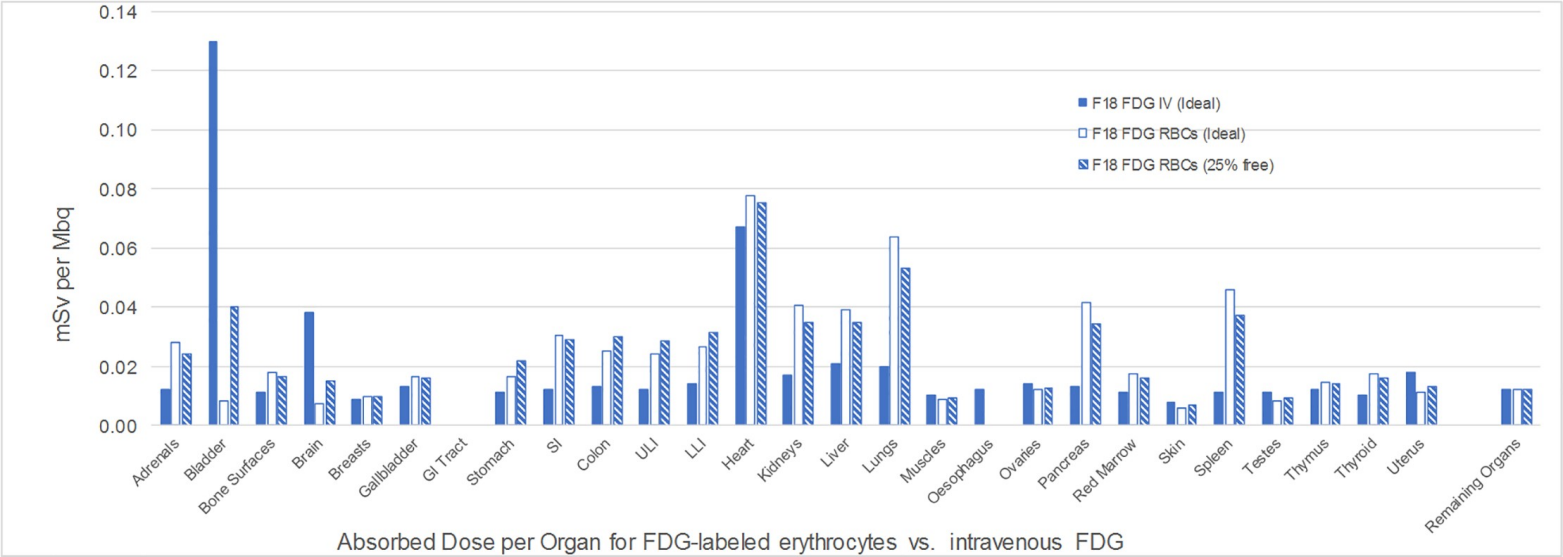
Comparison of calculated human organ dose from FDG-labeled erythrocytes with intravenous (IV) FDG. Organ dose estimates from FDG-labeled erythrocytes were calculated under conditions of 100% in vivo FDG retention and decay (FDG RBCs Ideal), and after 25% FDG excretion (FDG RBCs 25% free). In addition, organ dose estimates for intravenous FDG injection (FDG IV Ideal) were also calculated for comparison purposes.

Calculated human organ dose estimates from exposure to FDG-labeled erythrocytes were determined using the OLINDA v.1 software package. The organ absorbed doses resulting from the use of the OLINDA software and the idealized Christy-Eckerman phantom model are shown in **Table 1.** These idealized estimates were first calculated using the assumption that the dose to any given organ represents the fraction of the blood volume within the organ relative to the total blood pool, as it assumes that FDG-labeled erythrocytes distribute uniformly throughout the body vasculature. Under these conditions, we assume 100% tracer decay within the body with no urinary excretion of FDG. In addition, we also provide organ dosimetry estimates under hypothetical conditions in which there is poor FDG labeling of human erythrocytes with resulting 25% urinary excretion of free/released FDG (Fig 7). Organ dosimetry data was also calculated for intravenous FDG and ^99m^Tc labeled erythrocytes for comparative purposes.

The effective dose to the patient accounts for the relative susceptibility of the various organs to radiation damage and is estimated to be 3.90E-02 mSv per administered MBq using the tissue weighting factors outlined in ICRP Publication 60 [39] and used in OLINDA/EXM. The total effective dose to the patient from the injected radiopharmaceutical is expected to range from 7.2–14.4 mSv accounting for the planned injection range of 185–370 MBq (5-10mCi). In comparison, the expected effective doses to a patient receiving ^99m^Tc labeled erythrocytes (555–1110 MBq) or intravenous FDG (370–740 MBq) would be 3.89–7.77 mSv and 7–14 mSv, respectively, if the SNMMI Guidelines for administered doses are followed.

Per the dose limits outlined in 21 CFR 361.1 for both single dose administration and annual limits, the dose limiting organ is the heart wall [40]. During a single administration of F18-FDG labelled erythrocytes, the lungs and spleen receive the next two highest doses following the heart wall. For repeated administrations, the dose to the entire body reaches the regulatory limit prior to all organs other than the heart wall. Assuming no other radiation to the patient as part of the experimental imaging procedure, patients could receive a total of 5 administrations each year.

**Table 1:** Calculated organ absorbed doses using the OLINDA dosimetry software and Christy-Eckerman phantom.

Organs	Absorbed dose (mGy / MBq) Adult Male (No FDG leakage)	Absorbed dose (mGy / MBq) Adult Male (25% free FDG)
**Adrenals**	2.82E-02	2.42E-02
**Bladder**	9.70E-03	3.98E-02
**Bone Surfaces—Osteogenic Cells**	1.81E-02	1.63E-02
**Brain**	7.16E-03	1.49E-02
**Breasts**^a^	9.75E-03	9.51E-03
**Gallbladder**	1.64E-02	1.56E-02
**GI Tract**		
**Stomach**	2.48E-02	2.13E-02
**SI**	3.46E-02	2.90E-02
**Colon**^b^	3.52E-02	2.97E-02
**ULI**	3.37E-02	2.83E-02
**LLI**	3.67E-02	3.10E-02
**Heart**	7.78E-02	7.51E-02
**Kidneys**	4.07E-02	3.48E-02
**Liver**	3.94E-02	3.48E-02
**Lungs**	6.39E-02	5.29E-02
**Muscles**	8.71E-03	9.03E-03
**Ovaries**^a^	1.21E-02	1.26E-02
**Pancreas**	4.14E-02	3.43E-02
**Red Marrow**	1.77E-02	1.60E-02
**Skin**	5.93E-03	6.40E-03
**Spleen**	4.59E-02	3.72E-02
**Testes**^a^	8.44E-03	9.08E-03
**Thymus**	1.47E-02	1.40E-02
**Thyroid**	1.76E-02	1.57E-02
**Uterus**^a^	1.11E-02	1.28E-02
**Remaining Organs**	1.21E-02	1.21E-02

Left column: Target organ. Middle column: mGy/MBq in adult male under condition of no intracellular FDG leakage. Right column: mGy/MBq in adult male under condition of 25% initial FDG leakage.

^a^The Christy-Eckerman phantom is hermaphroditic and includes both male and female organs.

^b^Colon absorbed dose is an average of the doses to the upper (ULI) and lower (LLI) large intestine (S2 File).

## Discussion

Currently, ^99m^Tc-labeled compounds remain the dominant radionuclide-specific clinical blood pool imaging agents. In particular, ^99m^Tc-labeled erythrocytes are commonly used to non-invasively search for sites of occult lower intestinal bleeding and to measure cardiac contractility (left ventricular ejection fraction) in patients at risk for chemotherapy-induced cardiotoxicity. Given the inherent advantages of PET over gamma camera imaging, development of 18-fluorine-based blood pool imaging agents for these and other clinical applications remains an area of active research.

SPECT blood pool imaging has also been described in the medical literature, primarily in the setting of radionuclide ventriculography with 99m-Tc-labeled erythrocytes. While results comparable to that obtained with planar-based radionuclide ventriculography have been described, its use in the clinical setting is likely limited, as other imaging tests including myocardial perfusion SPECT can yield similar information [41]. Magnetic resonance blood pool imaging has also been described primarily with the use of the FDA-approved gadolinium contrast agent Gadofosveset; however, its current availability status from the manufacturer [Lantheus Medical Imaging] remains unknown.

Here, we take advantage of the high physiologic glucose uptake rate of FDG by human erythrocytes to show that human erythrocytes can be labeled with a sufficient amount of FDG for in vivo imaging of the vasculature of immunodeficient NSG mice with microPET/CT imaging. Rebinning of ECG-gated microPET images allowed visualization of the cardiac cycle in the mouse heart.

While erythrocytes collected ≤ 24 hours from the time of phlebotomy retained the ability to internalize significant amounts of FDG, we observed a negligible rate of internal FDG uptake when human erythrocytes are collected and used ≈ 5 days after phlebotomy, suggesting a significant drop in FDG uptake by glucose transporters and/or intracellular FDG metabolism in these somewhat older cells.

Importantly, FDG erythrocyte labeling was accomplished using a straightforward incubation and wash procedure that can be completed in a relatively short period of time (30 minute FDG incubation; total preparation time ≈ 60–70 minutes) and with minimal equipment. We demonstrate that the repeated cell centrifugation and wash steps in the FDG labeling protocol resulted in minimal cell membrane damage, at least as assessed by flow cytometric analysis with the cell viability/eryptosis markers calcein AM and Annexin V-PE. In addition, the simple incubation/wash buffer used in the protocol is composed of physiologic concentrations of a few salts, and a subclinical (≤1%) dose of the FDA approved chelating compound EDTA. These buffer constituents should thus not represent a significant impediment to clinical translation [42]. Our results also find that a single wash step leaves an acceptably small percentage of free FDG in the washed cell preparation. Based on our preclinical observations, we predict the above described FDG labeling/wash protocol should allow for up to ≈ 285 MBq of FDG incorporated in a volume of 10 ml’s packed erythrocytes, starting from an initial 740 MBq FDG amount. Furthermore, FDG uptake/metabolism appears dependent on time from phlebotomy; as such, freshly phlebotomized human blood may have an even higher FDG erythrocyte labeling efficiency.

One potential limitation to this technique is that there is a slow gradual release of intracellular FDG from erythrocytes over time. We have found that the relative FDG leakage fraction is approximate 11% at 46 minutes and 19% at 76 minutes. The relevance of the magnitude of FDG leakage would likely depend on the particular clinical application, but it would be reasonable to assume that the typical time course for clinical imaging using this technique would be up to 1 hour, with a resulting estimated leakage fraction of approximately 14%.

Recently, Matsusaka et al demonstrated that adult rat erythrocytes were able to incorporate FDG in vitro and could be used to image the body vasculature of rats using a microPET scanner. Our results are similar to those of the Matsusaka group, as they showed an estimated FDG-rat erythrocyte labeling efficiency of approximately 70% with their labeling procedure, and a low fraction (≈ 5–10%) of released FDG from erythrocytes after a 30 minute incubation period [43].

Given the lack of clinically significant adverse events associated with transfusion of either autologous erythrocytes or intravenous FDG, it is hypothesized that intravenous administration of FDG-labeled autologous human erythrocytes is unlikely to induce serious adverse clinical events. We have shown that repeated centrifugation and wash steps did not result in significant erythrocyte membrane damage. In addition, we did not detect a significant amount of urinary FDG excretion via PET imaging in these mice to suggest intravascular release of large amounts of intracellular FDG or byproducts from erythrocyte hemolysis or gross membrane leakage. It is also hypothesized that in the clinical setting, any mechanically damaged erythrocytes would be asymptomatically sequestered primarily in the spleen and the reticuloendothelial system, as this is the rationale for clinical imaging of patients with suspected/occult splenic tissue after injection of ^99m^Tc-labeled, heat-damaged autologous human erythrocytes [44].

Mice imaged with FDG-labeled human erythrocytes were previously splenectomized over concerns of potential xenogeneic-specific splenic sequestration of human erythrocytes. As a result, human erythrocytesequestration in the spleen of NSG mice would be difficult to accurately characterize. It is possible that similar imaging results can be obtained in mice with intact spleens; however, this remains to be further investigated by our group. As adult mouse erythrocytes have been found to have minimal membrane expression of GLUT1 transporters, mouse blood pool imaging using FDG-labeled mouse erythrocytes was not pursued to address this potential issue [29].

We found that all mice infused with human erythrocytes survived after imaging; however, some of the mice subjected to repeat injections of human erythrocytes experienced tachycardia and respiratory distress during and immediately after imaging that resolved 24 hours later. We speculate that this could be related to transient pulmonary/systemic edema from the relatively rapid injection of a large volume (≈500 microliters) of FDG-labeled erythrocyte suspension, despite prior retro-orbital plexus phlebotomy. It could also be related to hematopoietic engraftment of residual peripheral blood mononuclear cells contained in the first injection of human erythrocytes. Subsequent injection of human erythrocytes from a different human donor might have induced a type of allogeneic blood transfusion reaction in mice, although this remains somewhat speculative. This possibility could potentially be obviated in the future by using erythrocytes from a single human blood donor when repeated immunodeficient mice imaging is to be performed.

In addition, we present calculated human organ dosimetry data for FDG-labeled erythrocyte PET imaging using the OLINDA v.1 dosimetry software, with comparison made to organ doses from either 99m-Tc-labeled erythrocyte imaging or from intravenous FDG PET imaging. These idealized estimates were calculated using conditions where there is assumed to be either 100% tracer decay in the body with no FDG excretion, or under conditions where there is poor FDG labeling of human erythrocytes with resulting 25% urinary excretion of free/released FDG. As we have found that approximately 5% of the FDG in the labeled erythrocyte preparations is excreted into the mouse bladder over the imaging course of our in vivo experiments, we postulate that the calculated organ dosimetry range under these two theoretical conditions would provide reasonable guidance for translation to initial human clinical trials. Mouse organ dosimetry data was not collected, as the use of a splenectomized mouse model was expected to result in skewing of the relative blood volume distribution within the remaining mouse organs, and thus diminish the accuracy of any resulting organ dosimetry results for this blood pool imaging technique.

Our calculations show that the organ limiting dose from FDG-labeled erythrocyte imaging would be the myocardial wall; however, it should be noted that the calculated dose is only mildly higher than the dose received from intravenous FDG. As there are several organs where there is ≈ 2-3X higher organ dose compared to that from intravenous FDG (adrenals, bowel, kidneys, liver, lungs, pancreas, and spleen), patient exposure to 185–370 MBq of FDG-labeled erythrocytes would be predicted to have an overall dosimetry level somewhat comparable to that received from amounts of intravenous FDG used in clinical practice (370–740 MBq).

## Conclusion

We show that human erythrocytes can rapidly incorporate sufficient amounts of FDG to obtain in vivo images of the mouse vasculature using microPET/CT. We believe this imaging technique can be readily translated to the clinical setting, given the simplicity, high labeling efficiency, and relatively short turn-around time of the labeling protocol, as well as the use of a simple incubation/wash solution composed of a few physiologic salts and a subtherapeutic dose of a clinically used compound (EDTA). As modern medical PET/CT scanners generally possess count detection sensitivities much higher than that of medical gamma camera scintigraphy, FDG-erythrocyte PET imaging may have significant advantages over ^99m^Tc-labeled erythrocyte imaging, when used for the detection of either occult intestinal bleeding or chemotherapy-related cardiotoxicity. We speculate that this technique may have inherent advantages over the cerebrovascular perfusion SPECT imaging agents HMPAO and ECD for similar reasons. We also speculate that the relatively long radioactive half-life of 18F-FDG may allow for real-time detection of subtle changes in blood flow in the brain as well as other organs under varying conditions/stimuli that may not be discernable with clinical magnetic resonance imaging (MRI) platforms, due to the significantly higher sensitivity of PET over MRI [45].

## Supporting information

S1 Fig Flow cytometric analysis of erythrocyte membrane damage. Unperturbed sample 1.
(TIF)

S2 Fig Flow cytometric analysis of erythrocyte membrane damage. Unperturbed sample 2.
(TIF)

S3 Fig Flow cytometric analysis of erythrocyte membrane damage. Unperturbed sample 3.
(TIF)

S4 Fig Flow cytometric analysis of erythrocyte membrane damage. CaCl_2_-treated sample 1.
(TIF)

S5 Fig Flow cytometric analysis of erythrocyte membrane damage. CaCl_2_-treated sample 2.
(TIF)

S6 Fig Flow cytometric analysis of erythrocyte membrane damage. CaCl_2_-treated sample 3.
(TIF)

S7 Fig Flow cytometric analysis of erythrocyte membrane damage. Mock FDG-labeled sample 1.
(TIF)

S8 Fig Flow cytometric analysis of erythrocyte membrane damage. Mock FDG-labeled sample 2.
(TIF)

S9 Fig Flow cytometric analysis of erythrocyte membrane damage. Mock FDG-labeled sample 3.
(TIF)

S1 File Release of intracellular FDG from erythrocytes. Relative fraction of intracellular release of FDG from rat erythrocytes over time.
(XLSX)

S2 File OLINDA calculations. Calculated organ-specific absorbed dose per unit activity administered (mGy/MBq).
(XLSX)

## References

[1] Burow RD, Strauss HW, Singleton R, Pond M, Rehn T, Bailey IK, et al Analysis of left ventricular function from multiple gated acquisition cardiac blood pool imaging. Comparison to contrast angiography. Circulation. 1977 12;56(6):1024–8. . Epub 1977/12/01. eng. 923040 10.1161/01.cir.56.6.1024

[2] Thorne DA, Datz FL, Remley K, Christian PE. Bleeding rates necessary for detecting acute gastrointestinal bleeding with technetium-99m-labeled red blood cells in an experimental model. Journal of nuclear medicine: official publication, Society of Nuclear Medicine. 1987 4;28(4):514–20. . Epub 1987/04/01. eng. 3494826

[3] Wang ZG, Zhang GX, Hao SH, Zhang WW, Zhang T, Zhang ZP, et al Technological value of SPECT/CT fusion imaging for the diagnosis of lower gastrointestinal bleeding. Genetics and molecular research: GMR. 2015 11 24;14(4):14947–55. 10.4238/2015.November.24.2 . Epub 2015/12/05. eng. 26634456

[4] Groch MW, DePuey EG, Belzberg AC, Erwin WD, Kamran M, Barnett CA, et al Planar imaging versus gated blood-pool SPECT for the assessment of ventricular performance: a multicenter study. Journal of nuclear medicine: official publication, Society of Nuclear Medicine. 2001 12;42(12):1773–9. . Epub 2001/12/26. eng. 11752072

[5] Kim SJ, Kim IJ, Kim YS, Kim YK. Gated blood pool SPECT for measurement of left ventricular volumes and left ventricular ejection fraction: comparison of 8 and 16 frame gated blood pool SPECT. The international journal of cardiovascular imaging. 2005 Apr-Jun;21(2–3):261–6. 10.1007/s10554-004-6133-0 . Epub 2005/07/15. eng. 16015439

[6] Pelletier-Galarneau M, Finnerty V, Tan S, Authier S, Gregoire J, Harel F. Assessment of left ventricular ejection fraction with cardiofocal collimators: Comparison between IQ-SPECT, planar equilibrium radionuclide angiography, and cardiac magnetic resonance. Journal of nuclear cardiology: official publication of the American Society of Nuclear Cardiology. 2018 3 8 10.1007/s12350-018-1251-6 . Epub 2018/03/10. eng. 29520572

[7] Lewis M, Yanny S, Malcolm PN. Advantages of blood pool contrast agents in MR angiography: a pictorial review. Journal of medical imaging and radiation oncology. 2012;56(2):187–91. 10.1111/j.1754-9485.2012.02347.x 22498192

[8] Rahmim A, Zaidi H. PET versus SPECT: strengths, limitations and challenges. Nuclear medicine communications. 2008 3;29(3):193–207. 10.1097/MNM.0b013e3282f3a515 . Epub 2008/03/20. eng. 18349789

[9] Niu G, Lang L, Kiesewetter DO, Ma Y, Sun Z, Guo N, et al In Vivo Labeling of Serum Albumin for PET. Journal of nuclear medicine: official publication, Society of Nuclear Medicine. 2014 7;55(7):1150–6. 10.2967/jnumed.114.139642 . Pubmed Central PMCID: Pmc4576845. Epub 2014/05/21. Eng. 24842890 PMC4576845

[10] Basuli F, Li C, Xu B, Williams M, Wong K, Coble VL, et al Synthesis of fluorine-18 radio-labeled serum albumins for PET blood pool imaging. Nuclear medicine and biology. 2015 3;42(3):219–25. 10.1016/j.nucmedbio.2014.11.011 . Pubmed Central PMCID: Pmc4329020. Epub 2014/12/24. Eng. 25533724 PMC4329020

[11] Saatchi K, Gelder N, Gershkovich P, Sivak O, Wasan KM, Kainthan RK, et al Long-circulating non-toxic blood pool imaging agent based on hyperbranched polyglycerols. International journal of pharmaceutics. 2012 1 17;422(1–2):418–27. 10.1016/j.ijpharm.2011.10.036 . Epub 2011/11/03. Eng. 22044540

[12] de Langen AJ, van den Boogaart VE, Marcus JT, Lubberink M. Use of H2(15)O-PET and DCE-MRI to measure tumor blood flow. The oncologist. 2008 6;13(6):631–44. 10.1634/theoncologist.2007-0235 . Epub 2008/07/01. Eng. 18586918

[13] Fan AP, Jahanian H, Holdsworth SJ, Zaharchuk G. Comparison of cerebral blood flow measurement with [15O]-water positron emission tomography and arterial spin labeling magnetic resonance imaging: A systematic review. Journal of cerebral blood flow and metabolism: official journal of the International Society of Cerebral Blood Flow and Metabolism. 2016 5;36(5):842–61. 10.1177/0271678X16636393 . Pubmed Central PMCID: Pmc4853843. Epub 2016/03/06. Eng. 26945019 PMC4853843

[14] Ibaraki M, Miura S, Shimosegawa E, Sugawara S, Mizuta T, Ishikawa A, et al Quantification of cerebral blood flow and oxygen metabolism with 3-dimensional PET and 15O: validation by comparison with 2-dimensional PET. Journal of nuclear medicine: official publication, Society of Nuclear Medicine. 2008 1;49(1):50–9. 10.2967/jnumed.107.044008 . Epub 2007/12/14. Eng. 18077532

[15] Lodge MA, Jacene HA, Pili R, Wahl RL. Reproducibility of tumor blood flow quantification with 15O-water PET. Journal of nuclear medicine: official publication, Society of Nuclear Medicine. 2008 10;49(10):1620–7. 10.2967/jnumed.108.052076 . Pubmed Central PMCID: Pmc2587033. Epub 2008/10/04. Eng. 18832120 PMC2587033

[16] Green MA, Klippenstein DL, Tennison JR. Copper(II) bis(thiosemicarbazone) complexes as potential tracers for evaluation of cerebral and myocardial blood flow with PET. Journal of nuclear medicine: official publication, Society of Nuclear Medicine. 1988 9;29(9):1549–57. . Epub 1988/09/01. eng. 3261785

[17] Shelton ME, Green MA, Mathias CJ, Welch MJ, Bergmann SR. Assessment of regional myocardial and renal blood flow with copper-PTSM and positron emission tomography. Circulation. 1990 9;82(3):990–7. . Epub 1990/09/01. eng. 2394015 10.1161/01.cir.82.3.990

[18] Welch MJ, McCarthy TJ. The potential role of generator-produced radiopharmaceuticals in clinical PET. Journal of nuclear medicine: official publication, Society of Nuclear Medicine. 2000 2;41(2):315–7. . Epub 2000/02/25. eng. 10688117

[19] Haynes NG, Lacy JL, Nayak N, Martin CS, Dai D, Mathias CJ, et al Performance of a 62Zn/62Cu generator in clinical trials of PET perfusion agent 62Cu-PTSM. Journal of nuclear medicine: official publication, Society of Nuclear Medicine. 2000 2;41(2):309–14. . Epub 2000/02/25. eng. 10688116

[20] Wong TZ, Lacy JL, Petry NA, Hawk TC, Sporn TA, Dewhirst MW, et al PET of hypoxia and perfusion with 62Cu-ATSM and 62Cu-PTSM using a 62Zn/62Cu generator. AJR American journal of roentgenology. 2008 2;190(2):427–32. 10.2214/AJR.07.2876 . Pubmed Central PMCID: Pmc2980329. Epub 2008/01/24. eng. 18212229 PMC2980329

[21] Zhang T, Das SK, Fels DR, Hansen KS, Wong TZ, Dewhirst MW, et al PET with 62Cu-ATSM and 62Cu-PTSM is a useful imaging tool for hypoxia and perfusion in pulmonary lesions. AJR American journal of roentgenology. 2013 11;201(5):W698–706. 10.2214/AJR.12.9698 . Pubmed Central PMCID: Pmc4046635. Epub 2013/10/24. eng. 24147499 PMC4046635

[22] Flower MA, Zweit J, Hall AD, Burke D, Davies MM, Dworkin MJ, et al 62Cu-PTSM and PET used for the assessment of angiotensin II-induced blood flow changes in patients with colorectal liver metastases. European journal of nuclear medicine. 2001 1;28(1):99–103. . Epub 2001/02/24. eng. 11202458 10.1007/s002590000410

[23] Green MA, Mathias CJ, Welch MJ, McGuire AH, Perry D, Fernandez-Rubio F, et al Copper-62-labeled pyruvaldehyde bis(N4-methylthiosemicarbazonato)copper(II): synthesis and evaluation as a positron emission tomography tracer for cerebral and myocardial perfusion. Journal of nuclear medicine: official publication, Society of Nuclear Medicine. 1990 12;31(12):1989–96. . Epub 1990/12/01. eng. 2266398

[24] Wallhaus TR, Lacy J, Stewart R, Bianco J, Green MA, Nayak N, et al Copper-62-pyruvaldehyde bis(N-methyl-thiosemicarbazone) PET imaging in the detection of coronary artery disease in humans. Journal of nuclear cardiology: official publication of the American Society of Nuclear Cardiology. 2001 Jan-Feb;8(1):67–74. . Epub 2001/02/22. eng. 11182711 10.1067/mnc.2001.109929

[25] Herrero P, Hartman JJ, Green MA, Anderson CJ, Welch MJ, Markham J, et al Regional myocardial perfusion assessed with generator-produced copper-62-PTSM and PET. Journal of nuclear medicine: official publication, Society of Nuclear Medicine. 1996 8;37(8):1294–300. . Epub 1996/08/01. eng. 8708759

[26] Bailey DL, Eslick EM, Schembri GP, Roach PJ. (68)Ga PET Ventilation and Perfusion Lung Imaging-Current Status and Future Challenges. Seminars in nuclear medicine. 2016 9;46(5):428–35. 10.1053/j.semnuclmed.2016.04.007 . Epub 2016/08/25. eng. 27553468

[27] Bonte FJ, Hynan L, Harris TS, White CL 3rd. TC-99m HMPAO Brain Blood Flow Imaging in the Dementias with Histopathologic Correlation in 73 Patients. International journal of molecular imaging. 2011;2011:409101 10.1155/2011/409101 . Pubmed Central PMCID: Pmc3065903. Epub 2011/04/15. eng. 21490729 PMC3065903

[28] Goffin K, Dedeurwaerdere S, Van Laere K, Van Paesschen W. Neuronuclear assessment of patients with epilepsy. Seminars in nuclear medicine. 2008 7;38(4):227–39. 10.1053/j.semnuclmed.2008.02.004 . Epub 2008/06/03. eng. 18514079

[29] Montel-Hagen A, Blanc L, Boyer-Clavel M, Jacquet C, Vidal M, Sitbon M, et al The Glut1 and Glut4 glucose transporters are differentially expressed during perinatal and postnatal erythropoiesis. Blood. 2008 12 1;112(12):4729–38. 10.1182/blood-2008-05-159269 . Epub 2008/09/18. Eng. 18796630

[30] Viskupicova J, Blaskovic D, Galiniak S, Soszynski M, Bartosz G, Horakova L, et al Effect of high glucose concentrations on human erythrocytes in vitro. Redox biology. 2015 8;5:381–7. 10.1016/j.redox.2015.06.011 . Pubmed Central PMCID: Pmc4506982. Epub 2015/07/05. Eng. 26141922 PMC4506982

[31] Bratosin D, Mitrofan L, Palii C, Estaquier J, Montreuil J. Novel fluorescence assay using calcein-AM for the determination of human erythrocyte viability and aging. Cytometry Part A: the journal of the International Society for Analytical Cytology. 2005 7;66(1):78–84. . Epub 2005/05/26. eng. 15915509 10.1002/cyto.a.20152

[32] Pretorius E, du Plooy JN, Bester J. A Comprehensive Review on Eryptosis. Cellular physiology and biochemistry: international journal of experimental cellular physiology, biochemistry, and pharmacology. 2016;39(5):1977–2000. 10.1159/000447895 . Epub 2016/10/25. eng. 27771701

[33] Patlak CS, Blasberg RG, Fenstermacher JD. Graphical evaluation of blood-to-brain transfer constants from multiple-time uptake data. Journal of cerebral blood flow and metabolism: official journal of the International Society of Cerebral Blood Flow and Metabolism. 1983 3;3(1):1–7. 10.1038/jcbfm.1983.1 . Epub 1983/03/01. Eng. 6822610

[34] Valentin J. Basic anatomical and physiological data for use in radiological protection: reference values. A report of age- and gender-related differences in the anatomical and physiological characteristics of reference individuals. ICRP Publication 89. Annals of the ICRP. 2002;32(3–4):5–265. 14506981

[35] Stabin MG, Siegel JA. Physical models and dose factors for use in internal dose assessment. Health physics. 2003 9;85(3):294–310. . Epub 2003/08/27. eng. 12938720 10.1097/00004032-200309000-00006

[36] Stabin MG, Sparks RB, Crowe E. OLINDA/EXM: the second-generation personal computer software for internal dose assessment in nuclear medicine. Journal of nuclear medicine: official publication, Society of Nuclear Medicine. 2005 6;46(6):1023–7. . Epub 2005/06/07. eng. 15937315

[37] Christy M, Eckerman KF. Specific Absorbed Fractions of Energy at Various Ages from Internal Photon Sources. I. Methods. Oak Ridge, TN, USA: Metabolism and Dosimetry Research Group, Health and Safety Research Division, Oak Ridge National Laboratory;1987.

[38] Stabin M. Fundamentals of Nuclear Medicine Dosimetry. New York, NY, U.S.A: Springer; 2008.

[39] The 1990 Recommendations of the International Commission on Radiological Protection. ICRP Publication 60. Ann ICRP. 1991;21((1–3)):1–201. 2053748

[40] Radioactive drugs for certain research uses. In. 21 CFR 361.12016.

[41] Raja S, Mittal BR, Santhosh S, Bhattacharya A, Rohit MK. Comparison of LVEF assessed by 2D echocardiography, gated blood pool SPECT, 99mTc tetrofosmin gated SPECT, and 18F-FDG gated PET with ERNV in patients with CAD and severe LV dysfunction. Nuclear medicine communications. 2014 11;35(11):1156–61. 10.1097/MNM.0000000000000182 . Epub 2014/08/22. eng. 25144559

[42] Lamas GA, Boineau R, Goertz C, Mark DB, Rosenberg Y, Stylianou M, et al EDTA chelation therapy alone and in combination with oral high-dose multivitamins and minerals for coronary disease: The factorial group results of the Trial to Assess Chelation Therapy. American heart journal. 2014 7;168(1):37–44.e5. 10.1016/j.ahj.2014.02.012 . Pubmed Central PMCID: Pmc4069605. Epub 2014/06/24. Eng. 24952858 PMC4069605

[43] Matsusaka Y, Nakahara T, Takahashi K, Iwabuchi Y, Nishime C, Kajimura M, et al 18F-FDG-labeled red blood cell PET for blood-pool imaging: preclinical evaluation in rats. EJNMMI research. 2017 12;7(1):19 10.1186/s13550-017-0266-3 . Pubmed Central PMCID: Pmc5328895. Epub 2017/03/01. eng. 28244021 PMC5328895

[44] MacDonald A, Burrell S. Infrequently performed studies in nuclear medicine: Part 1. Journal of nuclear medicine technology. 2008 9;36(3):132–43; quiz 45. 10.2967/jnmt.108.051383 . Epub 2008/08/16. Eng. 18703616

[45] James ML, Gambhir SS. A molecular imaging primer: modalities, imaging agents, and applications. Physiological reviews. 2012 4;92(2):897–965. 10.1152/physrev.00049.2010 . Epub 2012/04/27. eng. 22535898

