# Supplementary figures and images for: In vivo positron emission tomographic blood pool imaging in an immunodeficient mouse model using 18F-fluorodeoxyglucose labeled human erythrocytes
Source: PLoS One. 2019 Jan 25;14(1):e0211012. doi: 10.1371/journal.pone.0211012 (PMC6347438; doi:10.1371/journal.pone.0211012)

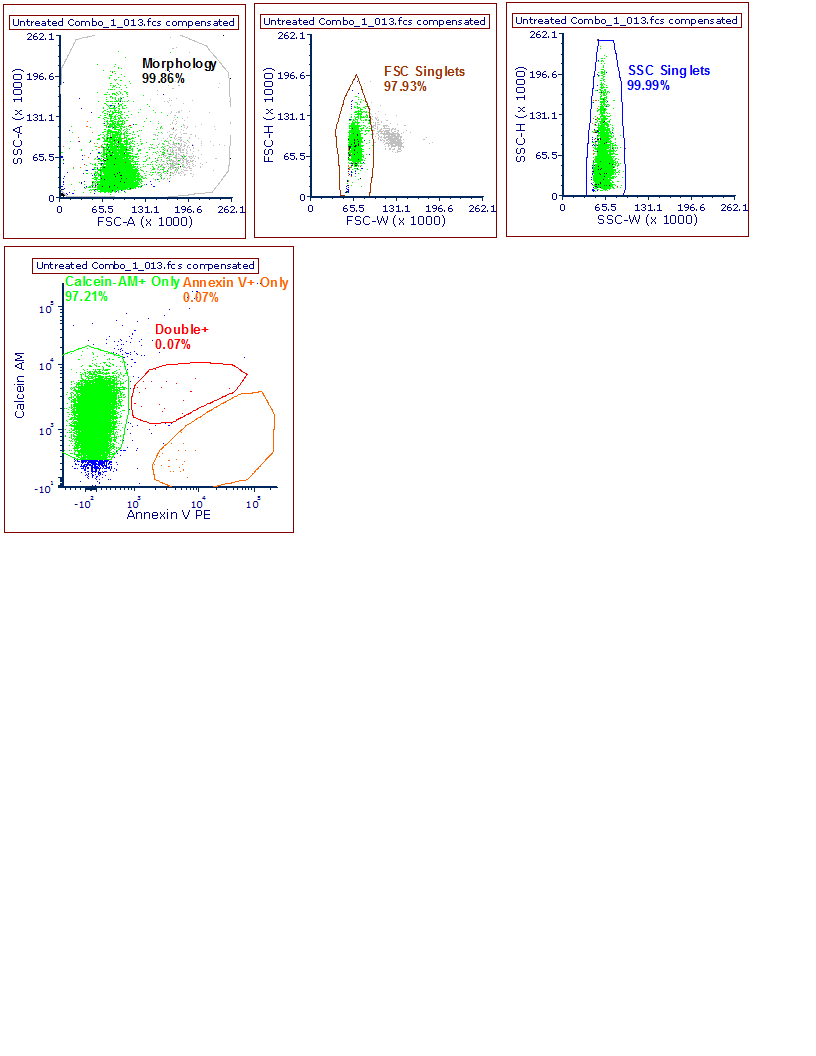

Supplement: S1 Fig — Unperturbed sample 1. (TIF) [file pone.0211012.s001.tif]

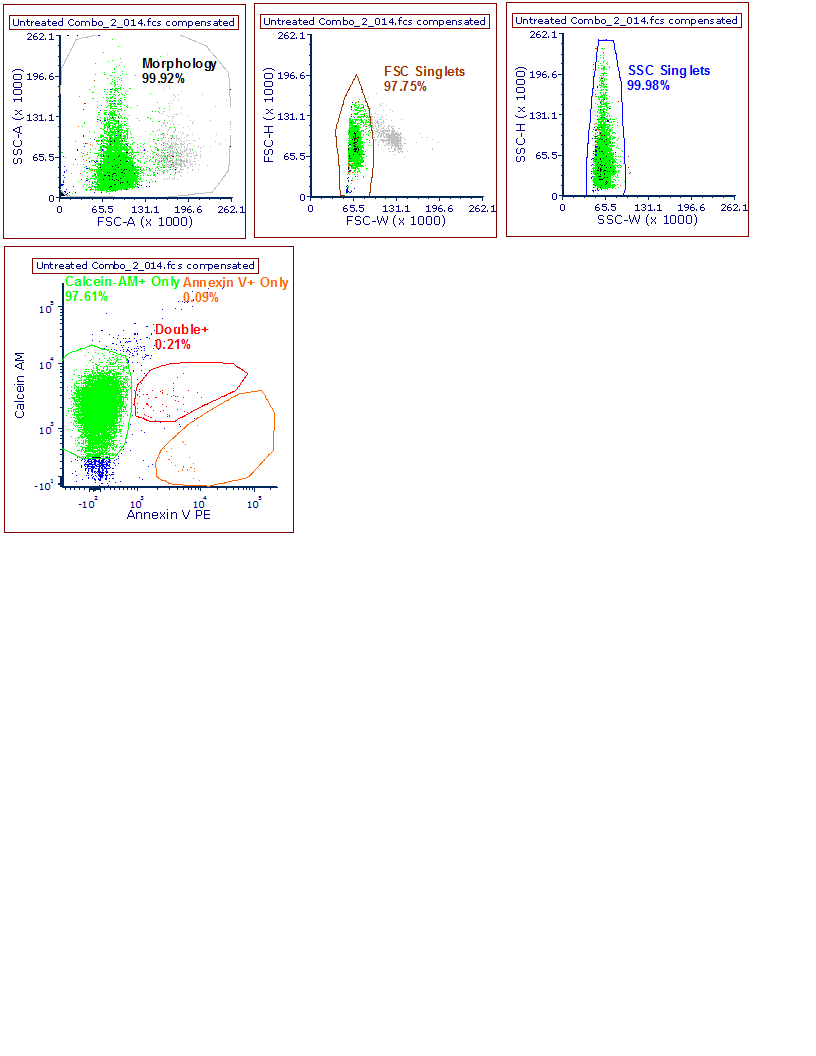

Supplement: S2 Fig — Unperturbed sample 2. (TIF) [file pone.0211012.s002.tif]

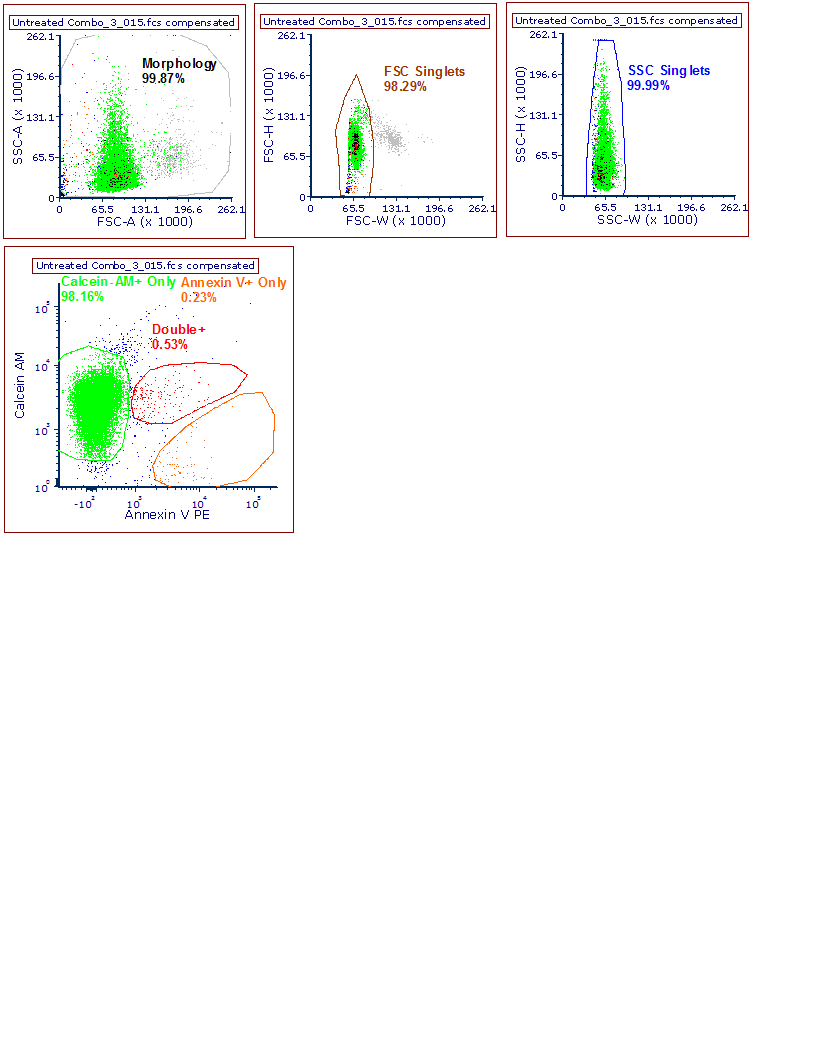

Supplement: S3 Fig — Unperturbed sample 3. (TIF) [file pone.0211012.s003.tif]

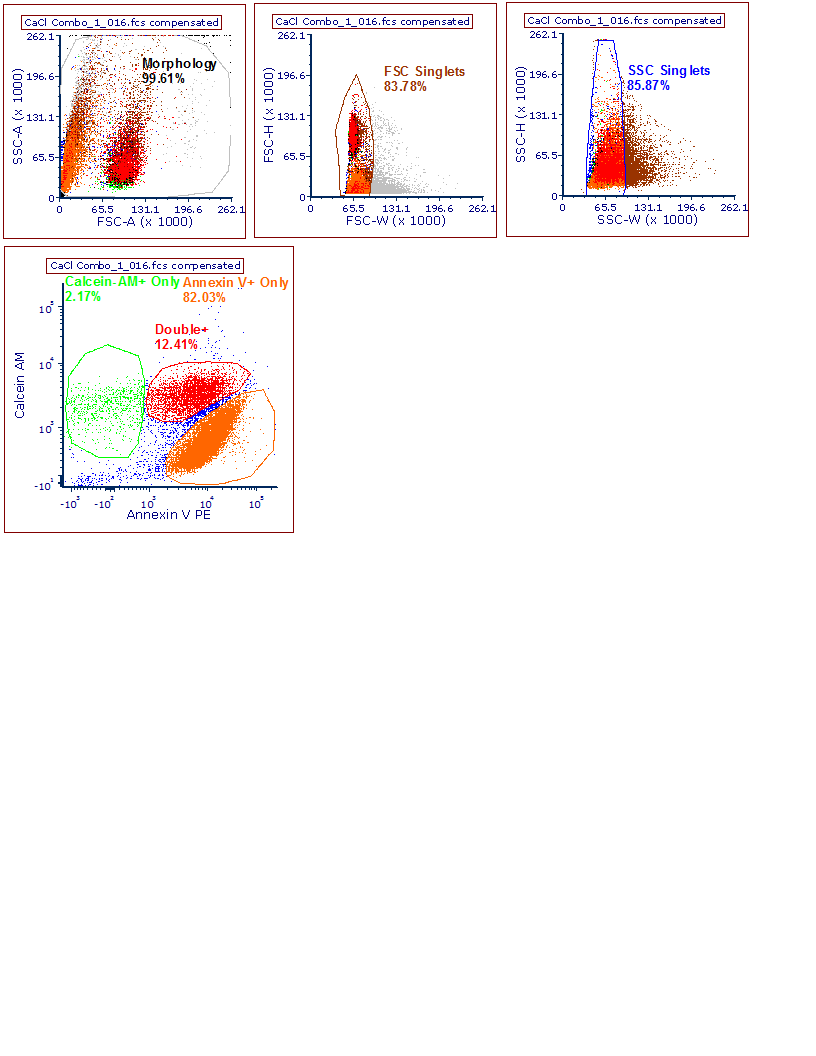

Supplement: S4 Fig — CaCl2-treated sample 1. (TIF) [file pone.0211012.s004.tif]

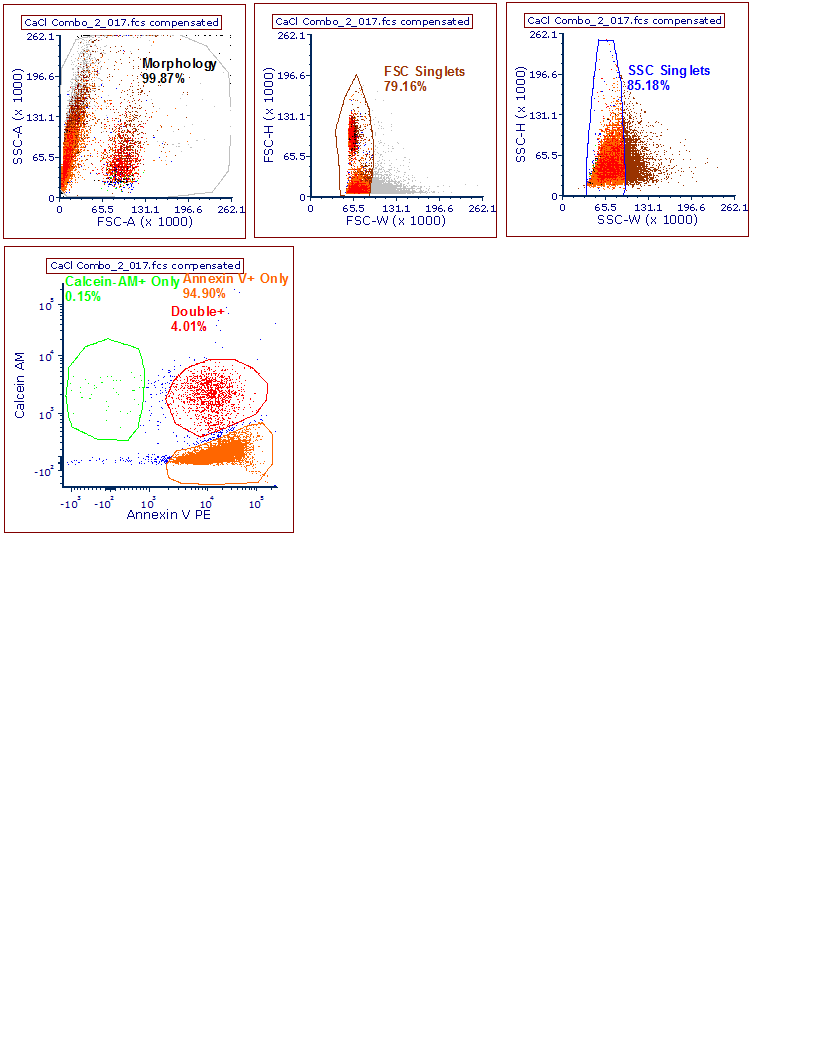

Supplement: S5 Fig — CaCl2-treated sample 2. (TIF) [file pone.0211012.s005.tif]

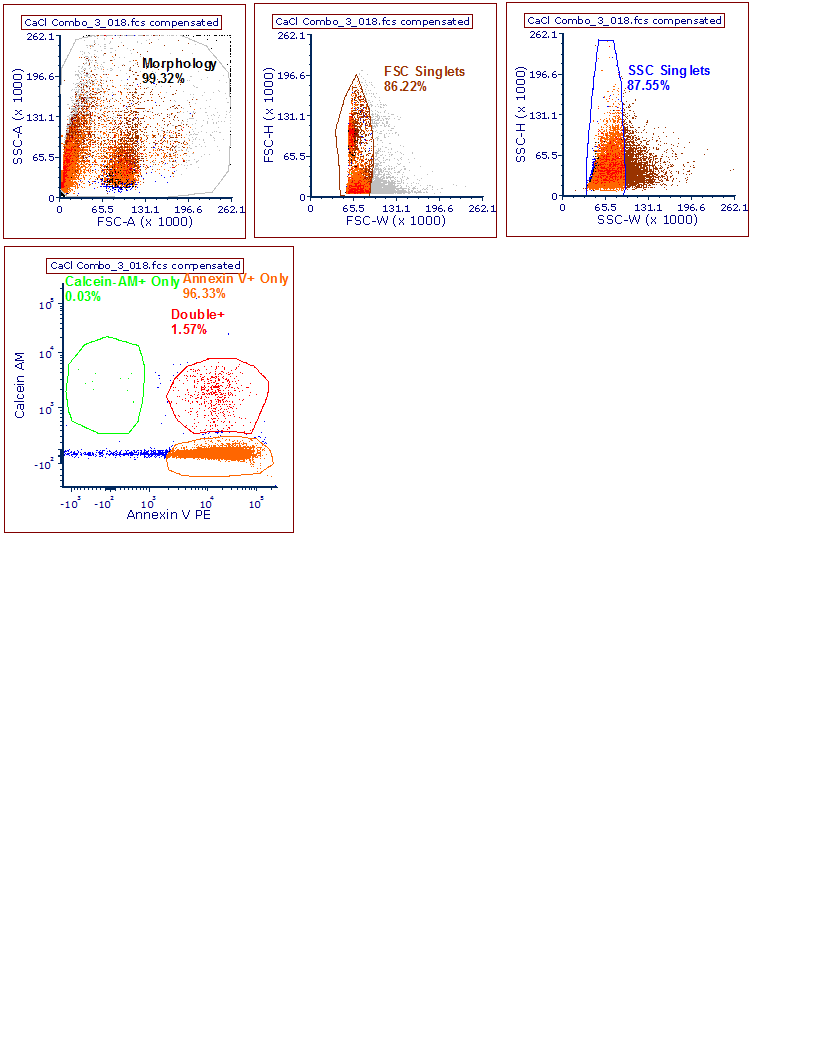

Supplement: S6 Fig — CaCl2-treated sample 3. (TIF) [file pone.0211012.s006.tif]

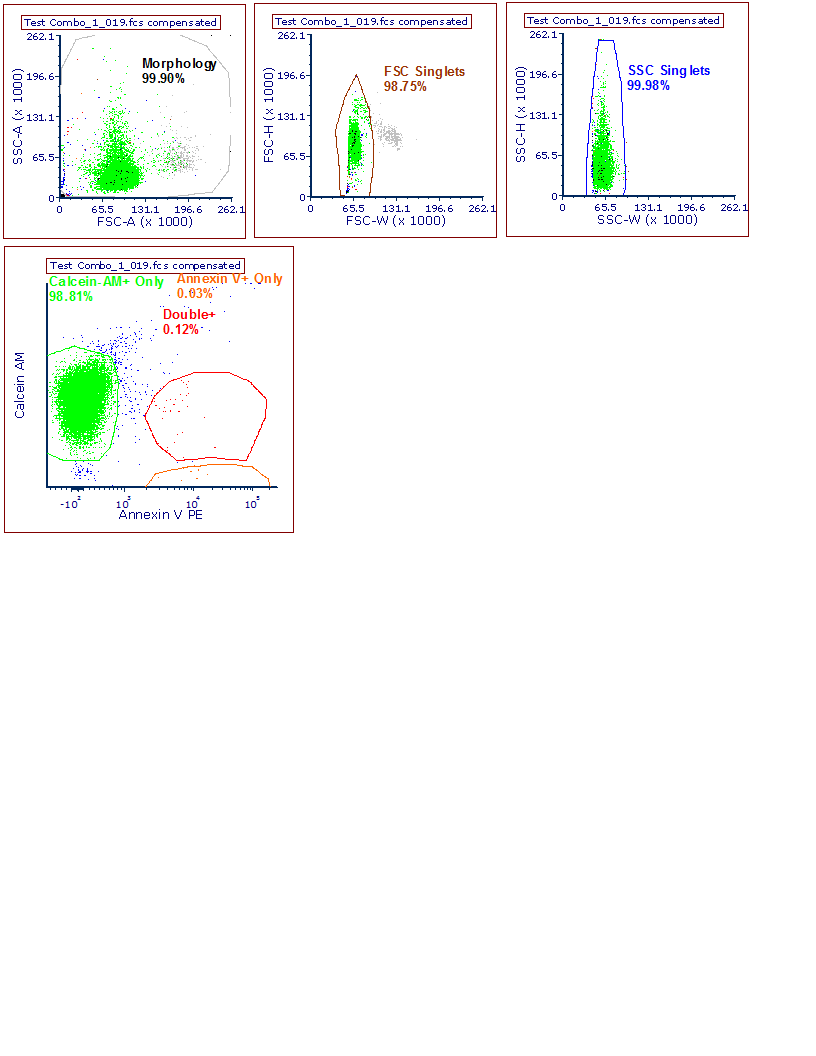

Supplement: S7 Fig — Mock FDG-labeled sample 1. (TIF) [file pone.0211012.s007.tif]

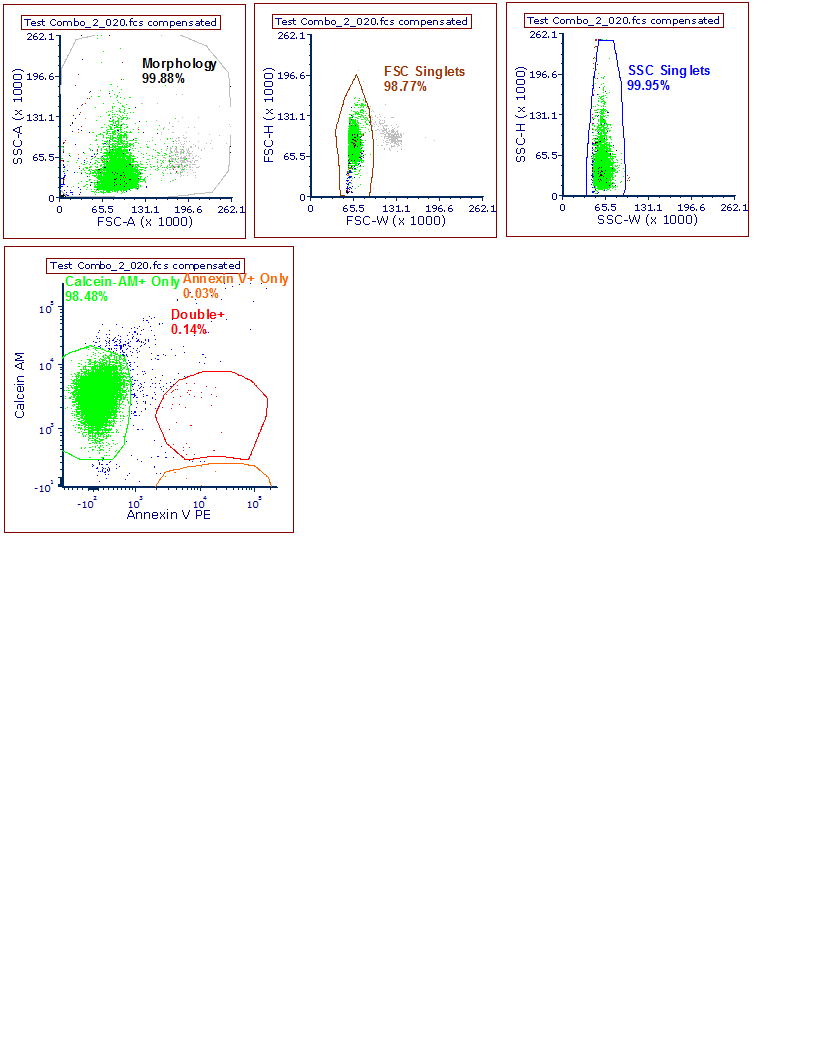

Supplement: S8 Fig — Mock FDG-labeled sample 2. (TIF) [file pone.0211012.s008.tif]

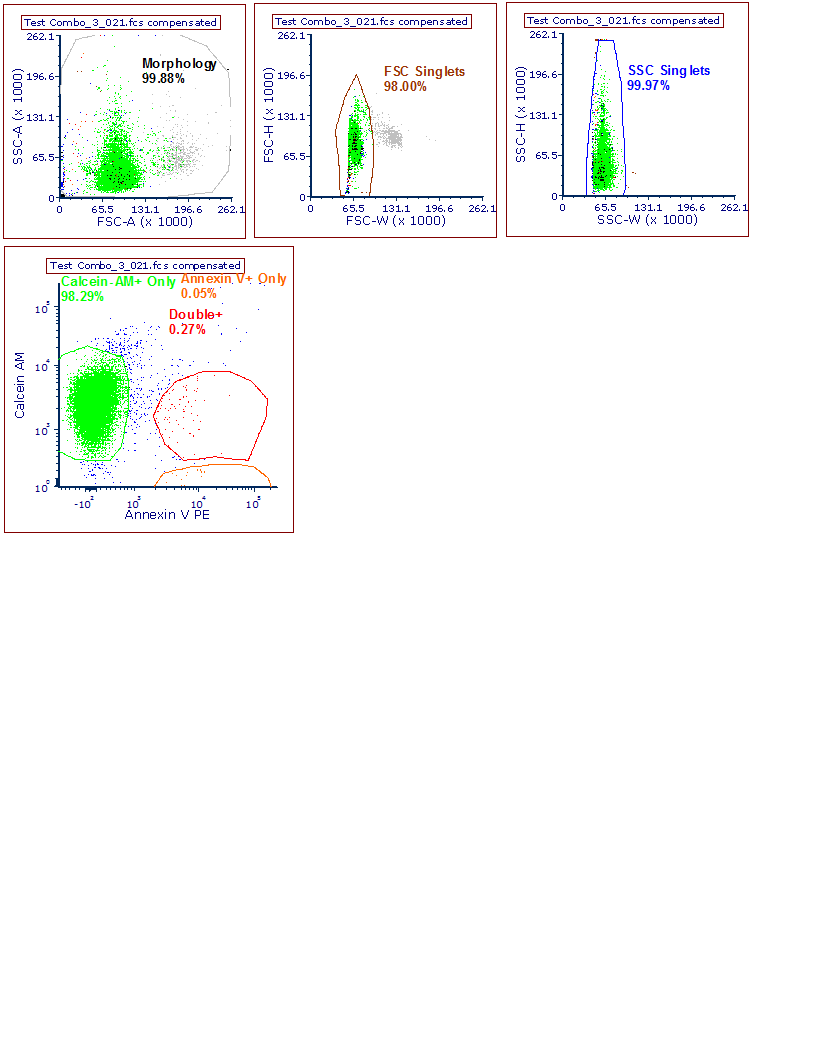

Supplement: S9 Fig — Mock FDG-labeled sample 3. (TIF) [file pone.0211012.s009.tif]
